# Correction: Abumaree et al. Decidua Basalis Mesenchymal Stem Cells Favor Inflammatory M1 Macrophage Differentiation In Vitro. *Cells* 2019, *8*, 173

**DOI:** 10.3390/cells15171606

**Published:** 2026-09-03

**Authors:** Mohamed H. Abumaree, Seham Al Harthy, Abdullah M. Al Subayyil, Manal A. Alshabibi, Fawaz M. Abomaray, Tanvier Khatlani, Bill Kalionis, Mohammed F. El- Muzaini, Mohammed A. Al Jumah, Dunia Jawdat, Abdullah O. Alawad, Ahmed S. AlAskar

**Affiliations:** 1 Stem Cells and Regenerative Medicine Department, King Abdullah International Medical Research Center, King Abdulaziz Medical City, Ministry of National Guard Health Affairs, P.O. Box 22490, Riyadh 11426, Saudi Arabiaalsubayyilab@ngha.med.sa (A.M.A.S.); khatlanita@ngha.med.sa (T.K.); jumahm@itkan-consulting.com (M.A.A.J.); jawdatd@ngha.med.sa (D.J.); askaras@ngha.med.sa (A.S.A.); 2 College of Science and Health Professions, King Saud Bin Abdulaziz University for Health Sciences, King Abdulaziz Medical City, Ministry of National Guard Health Affairs, P.O. Box 3660, Riyadh 11481, Saudi Arabia; 3 National Center for Stem Cell Technology, Life Sciences and Environment Research Institute, King Abdulaziz City for Science and Technology, P.O. Box 6086, Riyadh 11442, Saudi Arabia; ssalharthy@kacst.edu.sa (S.A.H.); malshabibi@kacst.edu.sa (M.A.A.); alawad@kacst.edu.sa (A.O.A.); 4 Department of Clinical Science, Intervention and Technology, Division of Obstetrics and Gynecology, Karolinska Institutet, 14186 Stockholm, Sweden; fawaz.abomaray@ki.se; 5 Center for Hematology and Regenerative Medicine, Karolinska Institutet, 14186 Stockholm, Sweden; 6 Department of Maternal-Fetal Medicine Pregnancy Research Centre and University of Melbourne, Department of Obstetrics and Gynaecology, Royal Women’s Hospital, Parkville, VIC 3052, Australia; 7 Department of Obstetrics and Gynaecology, King Abdulaziz Medical City, Ministry of National Guard Health Affairs, P.O. Box 3660, Riyadh 11481, Saudi Arabia; fmuzaini@hotmail.com; 8 College of Medicine, King Saud Bin Abdulaziz University for Health Sciences, King Abdulaziz Medical City, Ministry of National Guard Health Affairs, P.O. Box 3660, Riyadh 11481, Saudi Arabia; 9 Adult Hematology and Stem Cell Transplantation, King Abdulaziz Medical City, Ministry of National Guard Health Affairs, P.O. Box 22490, Riyadh 11426, Saudi Arabia

In the original publication [1], there was a mistake in Figure 1 as published. Figure 1D was inadvertently duplicated in Figure 1E. Figure 1 was originally compiled by the first author, Prof. Mohamed H. Abumaree, who unfortunately passed away after the paper was published. This tragic loss meant we could not verify the exact step that led to the assembly error, but we are confident it was an honest administrative mistake. The surviving co-authors searched the deceased author’s laboratory computer and physical notebooks. They retrieved the original, uncropped, and unedited images for the experiment. The co-authors matched the resolution, label, scale bar, and file format for the revised Figure 1E, as specified by the journal. The surviving authors reviewed the corrected Figure 1 and agreed to the change. The corrected Figure 1 appears below. The authors state that the scientific conclusions are unaffected. This correction was approved by the Academic Editor. The original publication has also been updated.

## Figures and Tables

**Figure 1:**
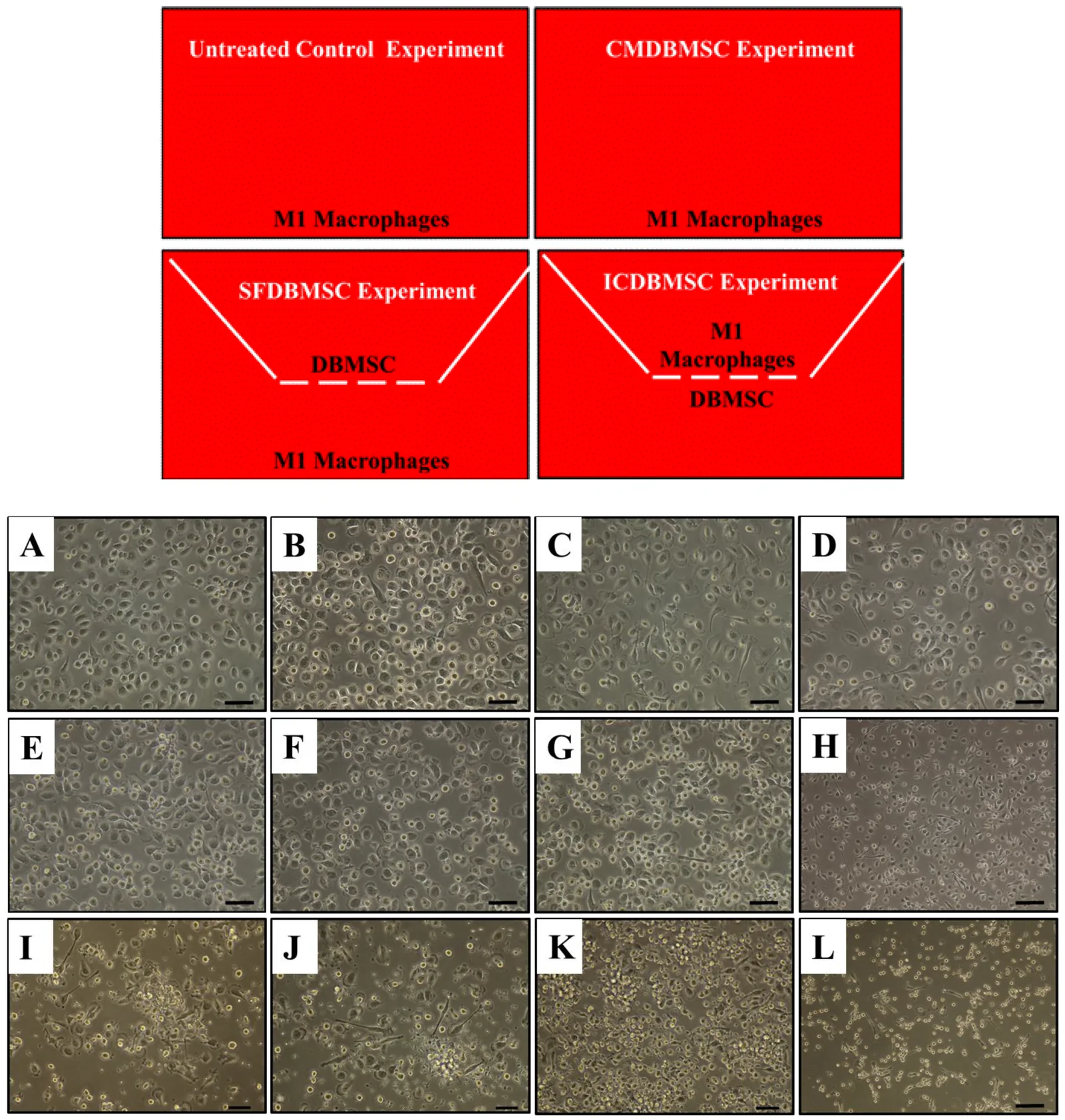
M1 macrophage culture system. Untreated control experiment consisted of M1 macrophages cultured on a surface of 6-well culture plate in a medium containing GM-CSF, CMDBMSC experiment consisted of M1 macrophages cultured on a surface of 6-well culture plate in a medium containing GM-CSF and CMDBMSC (conditioned medium), SFDBMSC (soluble factor), and ICDBMSC (intercellular direct contact) experiments. In SFDBMSC and ICDBMSC experiment, cells (DBMSCs and monocytes) were separated by transwell chamber membrane culture system. For the experiments of SFDBMSC, DBMSCs were seeded on the upper compartments while monocytes were seeded in the lower compartment. For the experiments of ICDBMSC, DBMSCs were seeded on the reverse side of the membrane while monocytes were seeded on the upper side of the membrane. GM-CSF medium was added to SFDBMSC and ICDBMSC experiments. Effects of human DBMSCs on the morphology of human monocytes differentiated into macrophages by GM-CSF. (**A**–**F**) Represent phase-contrast microscopic images showing monocyte (round-shaped morphology) differentiation into M1-like macrophages (fried egg-shaped morphology) after six days of culture in a medium containing GM-CSF (**A**), in a medium containing GM-CSF and DBMSCs at a 20:1 monocyte: DBMSC ratio (**B**), at a 10:1 monocyte: DBMSC ratio (**C**), at a 1:1 monocyte: DBMSC ratio (**D**), in the presence of 10% CMDBMSC (**E**), or in the presence of 20% CMDBMSC (**F**). (**G**–**K**) Representative phase-contrast microscopic images showing monocyte-like cells after six days of culture in a medium containing GM-CSF and 30% CMDBMSC (**G**), 40% CMDBMSC (**H**), 50% CMDBMSC (**I**), 60% CMDBMSC (**J**), 80% CMDBMSC (**K**), or 100% CMDBMSC (**L**). Experiments were carried out in duplicate and repeated 30 times using 30 individual preparations of both monocyte-derived macrophages and DBMSCs. Scale bars represent 50 µm.
